# Supplementary material for: Sliding nanomechanical resonators
Source: Nat Commun. 2022 Oct 27;13:6392. doi: 10.1038/s41467-022-34144-5 (PMC9613885; doi:10.1038/s41467-022-34144-5)
Supplement: Supplementary file 1 — Supplementary Information [file 41467_2022_34144_MOESM1_ESM.pdf]

Supplementary Information

for

Sliding nanomechanical resonators

Yue Ying[1,2†], Zhuo-Zhi Zhang[1,2†], Joel Moser[3,4*], Zi-Jia Su[1,2], Xiang-Xiang Song[1,2*], and Guo-Ping Guo[1,2,5*]

1. CAS Key Laboratory of Quantum Information, University of Science and Technology of China, Hefei, Anhui 230026, China
2. CAS Center for Excellence in Quantum Information and Quantum Physics, University of Science and Technology of China, Hefei, Anhui 230026, China
3. School of Optoelectronic Science and Engineering, Soochow University, Suzhou, Jiangsu 215006, China
4. Key Lab of Advanced Optical Manufacturing Technologies of Jiangsu Province, Soochow University, Suzhou, Jiangsu 215006, China
5. Origin Quantum Computing Company Limited, Hefei, Anhui 230088, China

† Y. Y. and Z.-Z. Z. contributed equally to this work.

* Author to whom correspondence should be addressed: J. M. (j.moser@suda.edu.cn), X.-X. S. (songxx90@ustc.edu.cn), or G.-P. G. (gpguo@ustc.edu.cn).

S1. Transfer characteristic curve

Figure S1 shows the transfer characteristic curves (here the output is dc current I_{dc}) of the device presented in the main text and obtained at dc source-drain bias $V_{\mathrm{dc}} = 300\,\mathrm{mV}$ for increasing and decreasing gate voltage V_{G}. Open blue circles represent the data for increasing V_{G} (labelled "upward"), which are directly measured. Solid red squares represent the data for decreasing V_{G} (labelled "downward"); these are extracted from a series of output characteristic curves measured at different V_{G}. In the "downward" direction, V_{G} is changed monotonically and all the data are collected within 20 minutes. Although the data for the "downward" direction have a larger dispersion than those for the "upward" direction, no obvious electrical hysteretic behavior is observed. We therefore rule out the possibility of electrical hysteresis in our device [S1].

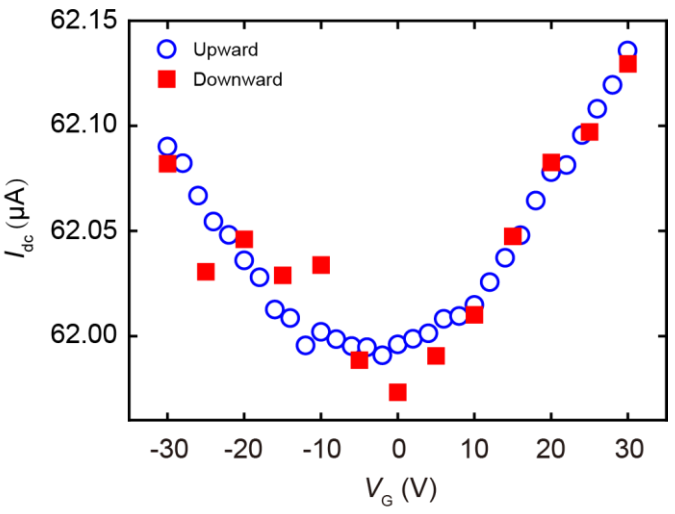

Fig. S1. Measured transfer characteristic curves for increasing V_{G} ("upward") and decreasing V_{G} ("downward") with $V_{\mathrm{dc}} = 300\,\mathrm{mV}$.

S2. Definition of stepping rate

The stepping rate is defined as the averaged rate for stepping gate voltage V_{G} to obtain a frequency loop. For example, the leftmost panel in Fig. 3a is made by superimposing two frequency spectra. For each spectrum, V_{G} changes by 10 V and the total time spent is 7715 s. Thus the stepping rate is 10 V/7715 s = 0.0013 V s^{-1}.

S3. Width of frequency loop as a function of gate voltage step size

We investigate the width of the frequency loop as a function of gate voltage step size V_{step}.

The latter is chosen to be 0.05 V, 0.1 V, and 0.2 V, while keeping the stepping rate constant at 0.0013 V s^{-1}. Figure S2 shows that the width of frequency loop does not noticeably depend on V_{step}.

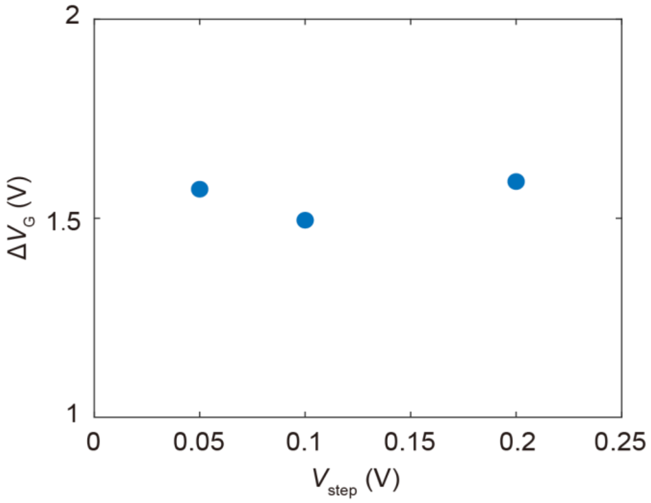

Fig. S2. Width of hysteresis loop ΔV_{G} obtained at different gate voltage step sizes V_{step} while keeping the stepping rate constant.

S4. Power dependence of frequency loops

We investigate frequency loops upon varying gate voltage V_{G} between 25 V and 30 V at different microwave drive powers (Fig. S3a). Measured spectra of electromechanical current I, along with fits to the theoretical response [S2, S3], near resonant frequency and at different drive powers are shown in Fig. S3b. Peaks in $I(f_{\text{d}})$ are clearly observed, where f_{d} is the drive frequency.

Figure S3 shows that changing the drive power does not change the shape of the frequency loop. In addition, the resonances are symmetrical. These results suggest that the Duffing mechanical nonlinearity is negligible and is unlikely to be the origin of the frequency loops [S4, S5].

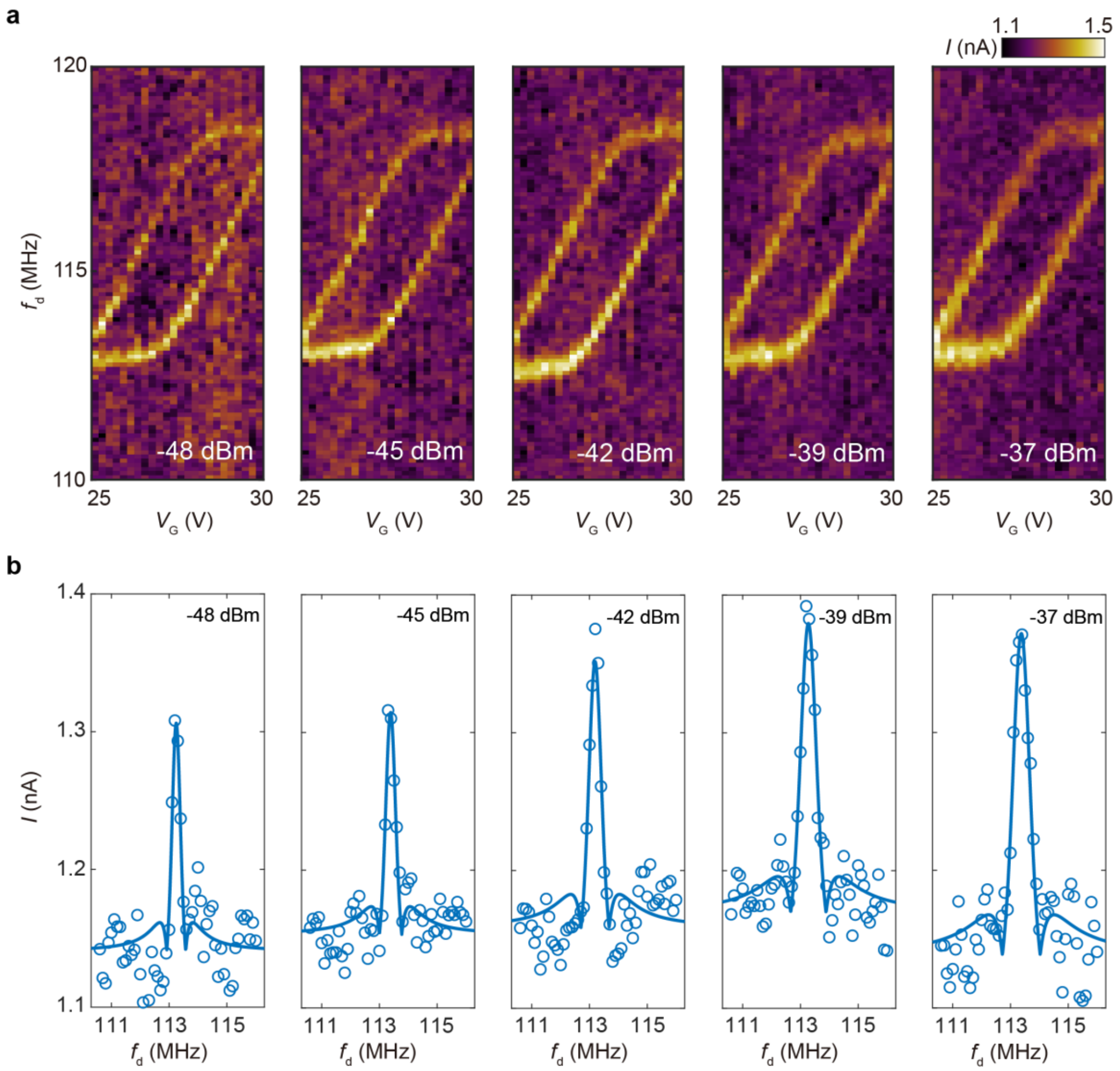

Fig. S3. (a) Frequency loops at different drive powers. The shape of the loops remains practically unchanged. (b) Measured mixing current spectra (open circles) and fits to theory (solid curves) at different drive powers, with V_G set to 27 V in the "upward" direction.

S5. Simulating gate capacitance

Based on experimental parameters, we perform COMSOL simulations to calculate the relation between the gate capacitance C and the equilibrium position z_e of the graphene flake. As shown in Fig. S4, the first derivative of the capacitance $C' = \frac{dC}{dz_e}$ is estimated to be $C' = 1.1 \times 10^{-9}$ F m^{-1}.

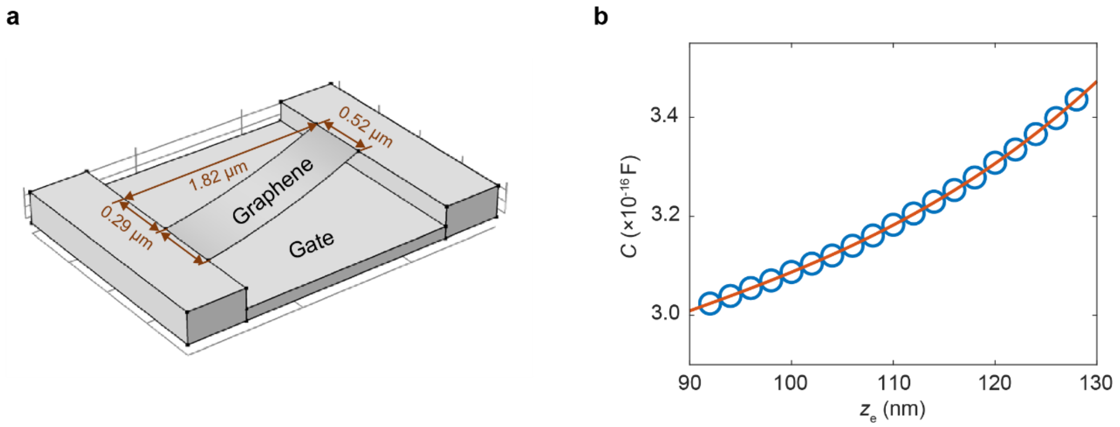

Fig. S4. (a) Schematic of the device used for COMSOL simulation. (b) Gate capacitance C as a function of equilibrium position z_e. The orange curve is the second-order polynomial fit of the simulated results (open blue circles).

S6. Viscoelasticity model

We consider a pre-tensioned, suspended string resonator with fixed boundary conditions. The resonator is made of a viscoelastic material (for example, fabrication residues) and incorporates a Voigt-Kelvin constitutive relationship [S6].

The total mechanical energy of the string resonator can be written as:

$$E_\mathrm{tot} = \frac{1}{2}S\sigma \cdot L\epsilon \tag{S6.1}$$

where ϵ and σ are the strain and stress in the string resonator, respectively. S is the cross-sectional area, and L is the length of the trench between the two contact electrodes.

Since ϵ is proportional to the elongation of the string, assuming the mode shape has a parabolic form we have:

$$L(\epsilon - \epsilon_0) = \frac{1}{4}\frac{x^2}{L} \tag{S6.2}$$

where ϵ_0 is the built-in strain and x is the deformation of the string along the vertical direction.

The electrostatic force F_elec between the gate electrode and the resonator, and the restoring force F_res, are:

$$F_\mathrm{elec} = \frac{1}{2}C'V_\mathrm{G}^2 \tag{S6.3}$$

$$F_\mathrm{res} = \frac{\partial E_\mathrm{tot}}{\partial x} = \frac{1}{4}\frac{S}{L}x \cdot \sigma \tag{S6.4}$$

Since $F_\mathrm{elec} = F_\mathrm{res}$, we have:

$$\frac{1}{4}\frac{S}{L}x\cdot\sigma=\frac{1}{2}C'V_{\mathrm{G}}^2 \tag{S6.5}$$

where V_{G} is the gate voltage and C' is the first derivative of the gate capacitance C with respect to displacement in the vertical direction.

Considering that the viscoelastic material incorporates a Voigt-Kelvin constitutive relationship, we have:

$$\sigma=E\epsilon+\eta\frac{\mathrm{d}\epsilon}{\mathrm{d}t} \tag{S6.6}$$

where E is the Young's modulus and η is the viscosity coefficient.

Plugging Eqs. (S6.6) and (S6.2) into Eq. (S6.5) yields:

$$S\sqrt{\epsilon-\epsilon_0}\times\left(E\epsilon+\eta\frac{\mathrm{d}\epsilon}{\mathrm{d}t}\right)=C'V_{\mathrm{G}}^2 \tag{S6.7}$$

that is

$$\frac{\mathrm{d}\epsilon}{\mathrm{d}t}=A\frac{V_{\mathrm{G}}^2}{\sqrt{\epsilon-\epsilon_0}}-B\epsilon \tag{S6.8}$$

where $A=\frac{C'}{\eta S}$ and $B=\frac{E}{\eta}$ are positive fit coefficients.

By solving Eq. (S6.8), we can calculate ϵ as a function of time t. The resonant frequency of the device can also be obtained:

$$f=2\pi\sqrt{\frac{1}{m_{\mathrm{eff}}}}\times\sqrt{\frac{\partial^2E_{\mathrm{tot}}}{\partial x^2}}=F\times\sqrt{6\epsilon-4\epsilon_0+\frac{1}{B}\frac{\mathrm{d}\epsilon}{\mathrm{d}t}} \tag{S6.9}$$

where m_{eff} is the effective mass of the mode. For example, we consider the case where $A=2\times10^{-9}\ \mathrm{V^{-2}\ s^{-1}}$, $B=5.1\times10^{-3}\ \mathrm{s^{-1}}$, $\epsilon_0=10^{-3}$, $F=8\times10^8$ Hz and V_{G} varies from 20 V to 25 V. The numerical solution to Eq. (S6.8) for a stepping rate of 0.01 V $\mathrm{s^{-1}}$ is shown in Fig. S5a. The corresponding frequency loop obtained from Eq. (S6.9) is shown in Fig. S5b. Although a loop can form, no plateau is found when the V_{G} stepping direction is reversed, as shown in the inset to Fig. S5b. The shape of the frequency loop is different from the experimental results.

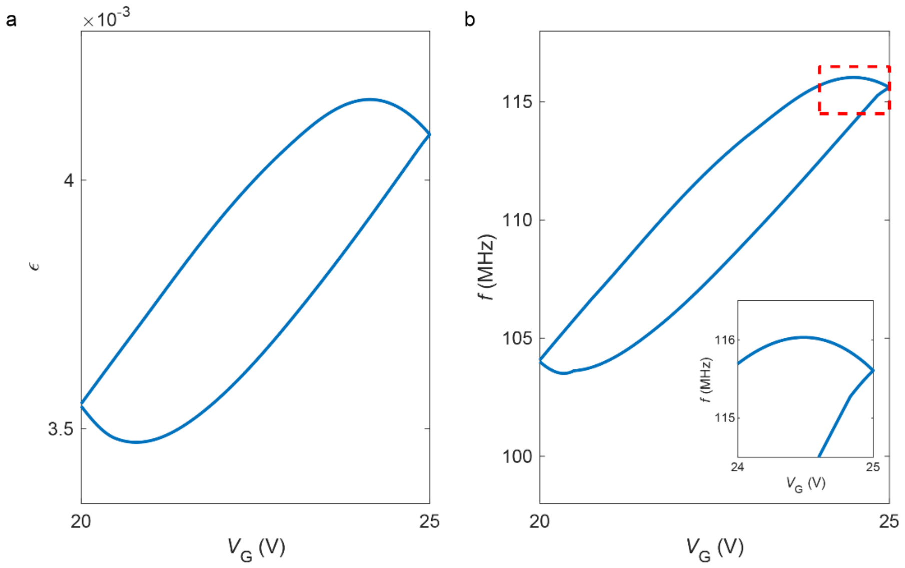

Fig. S5. (a) Strain ϵ and (b) resonant frequency f as a function of gate voltage V_G for $A = 2 \times 10^{-9}\ \mathrm{V}^{-2}\ \mathrm{s}^{-1}$, $B = 5.1 \times 10^{-3}\ \mathrm{s}^{-1}$, $F = 8 \times 10^{8}$ Hz, $\epsilon_0 = 10^{-3}$ and for a stepping rate of 0.01 V s^{-1}. The inset to (b) details the area within the red dashed box.

S7. Sliding model

Introduction to the model

We model a suspended graphene flake as a pre-tensioned, suspended string resonator connected to a rigid substrate via a viscoelastic connector (Fig. S6). The length of the trench between the two contact electrodes is L, the elongation of the connector is q, the built-in strain is ϵ_0, and the tension in the string is T. When the graphene flake deforms under an electrostatic force F, the connector also deforms due to changes in tension. As a result, the graphene flake slides on the supporting substrate and feeds extra length of the flake into the suspending part, thus changing the resonant frequency.

To quantitatively model the sliding process at the contact area, we assume that part of the string is a "contact string" of length L_1 at both contact electrodes. The "contact strings" connect the viscoelastic connectors (blue boxes in Fig. S6, consisting of a spring and a dashpot [S7]) to the suspended string. The strain and Young's modulus of the "contact strings" are the same as those of the suspended part. Also, the "contact strings" have no elastic or viscous interaction with the connector. Comparing the deformation of the viscoelastic connector q, the length of the trench between the two contact electrodes L and the length of each "contact string" L_1, we have $q <$

$L_1 \ll L$.

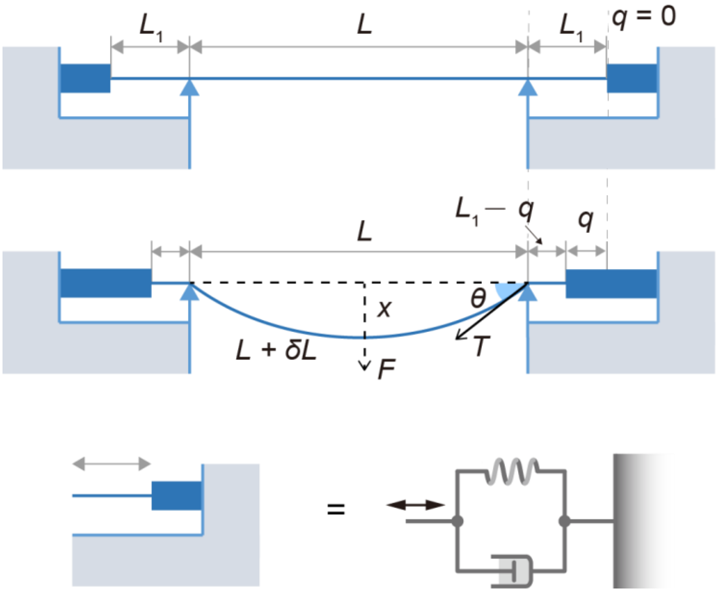

Fig. S6. Schematic describing the sliding model.

Mode shape

In our model, the graphene membrane is modeled as a 1-D string. The direction of internal tension must be the same as the direction of the string (see Fig. S7). So the mode shape of the suspended part is such that:

$$\xi'' \propto F_{\mathrm{elec}} \tag{S7.1}$$

where $\xi(y)$ is the mode shape, y is the horizontal coordinate, $\xi' = \mathrm{d}\xi/\mathrm{d}y, \xi'' = \mathrm{d}\xi'/\mathrm{d}y$, and F_{elec} is the electric force induced by the gate. We assume a uniform distribution of F_{elec}, *i.e.*, $\mathrm{d}F_{\mathrm{elec}}/\mathrm{d}y \equiv 0$.

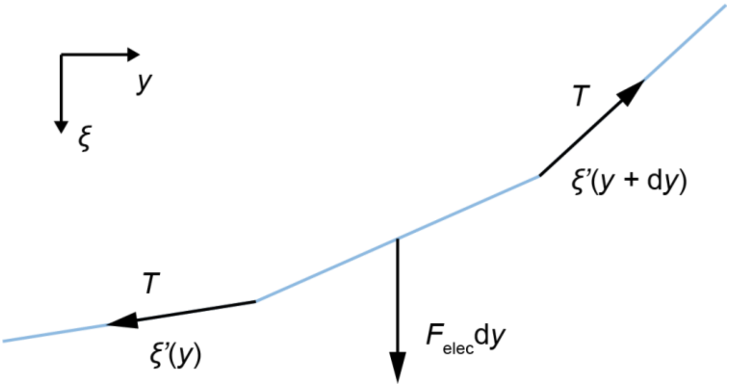

Fig. S7. Calculation of the mode shape.

We assume that, although the flake can slide on the contact electrodes, the suspended part of the flake is a quasi-static doubly-clamped string. The boundary conditions for the doubly-clamped string are:

$$\xi(0) = \xi(L) = 0 \tag{S7.2}$$

The solution for the mode shape has a parabolic form:

$$\xi(y) = x \cdot \frac{4}{L^2} y(L - y) \tag{S7.3}$$

where x is the maximum static displacement of the string. Thus we assume that the mode shape always has a parabolic form while x changes when the gate voltage changes.

Equilibrium equations

The dynamics of the system is determined by two geometric parameters: q and x. Below, we derive a set of coupled equations to describe their relationship with time-dependent gate voltage.

Assuming the original length of the string (including "suspended string" and "contact string") is L_0, the built-in strain ϵ_0 is:

$$\epsilon_0 = \frac{L + 2L_1 - L_0}{L_0} \tag{S7.4}$$

As shown in Fig. S6, the length of the string after deformation is $L_{\text{suspended}} + 2(L_1 - q)$.

Here the length of the deformed graphene string in the suspended part is:

$$L_{\text{suspended}} = \int_0^L \frac{\mathrm{d}y}{\cos(\theta)} \approx \int_0^L \left(1 + \frac{1}{2}\xi'^2\right) \mathrm{d}y = L + \frac{1}{4}\frac{x^2}{L} \tag{S7.5}$$

where θ is the angle between the string and the horizontal direction.

This yields the tension T as a function of x and q:

$$T = ES \frac{L + \frac{1}{4}\frac{x^2}{L} + 2(L_1 - q) - L_0}{L_0} \tag{S7.6}$$

where E is the Young's modulus and S is the cross-sectional area.

According to the Voigt-Kelvin constitutive relationship of the viscoelastic connector, the equilibrium equation at the connection point between the "contact string" and the viscoelastic connector is:

$$T = kq + c\frac{\mathrm{d}q}{\mathrm{d}t} \tag{S7.7}$$

Plugging Eqs. (S7.4) and (S7.6) into Eq. (S7.7), we have:

$$\frac{\mathrm{d}q}{\mathrm{d}t} = -\left(\frac{k}{c} + \frac{2ES}{cL_0}\right) q + \frac{1}{4}\frac{ES}{cLL_0} x^2 + \frac{ES}{c}\epsilon_0 \tag{S7.8}$$

The equilibrium equation for the deflection x of the suspended part is:

$$m_{\mathrm{eff}}\ddot{x} = F - 2T\sin(\theta) = F - 2T \times 4\frac{x}{L} \tag{S7.9}$$

where $F = \frac{1}{2}C'V_{\mathrm{G}}^2$ is the vertical force.

Plugging Eqs. (S7.4) and (S7.6) into Eq. (S7.9), we have:

$$\frac{\mathrm{d}^2x}{\mathrm{d}t^2} = \frac{C'V_{\mathrm{G}}^2}{2m_{\mathrm{eff}}} - \frac{8ES}{m_{\mathrm{eff}}L}\left(\frac{1}{4}\frac{x^2}{LL_0} - 2\frac{q}{L_0} + \epsilon_0\right)x \tag{S7.10}$$

Since $q < L_1 \ll L$, $\epsilon \ll 1$, we have $L_0 \approx L$. We can then replace L_0 in Eqs. (S7.8) and (S7.10) by L:

$$\frac{\mathrm{d}q}{\mathrm{d}t} = -\left(\frac{k}{c} + 2\frac{ES}{cL}\right) q + \frac{1}{4}\frac{ES}{cL^2} x^2 + \frac{ES}{c}\epsilon_0 \tag{S7.11}$$

$$\frac{\mathrm{d}^2x}{\mathrm{d}t^2} = \frac{C'V_{\mathrm{G}}^2(t)}{2m_{\mathrm{eff}}} - \frac{8ES}{m_{\mathrm{eff}}L}\left(\frac{1}{4}\frac{x^2}{L^2} - 2\frac{q}{L} + \epsilon_0\right)x \tag{S7.12}$$

Equations (S7.11) and (S7.12) determine how the system evolves with time-dependent gate voltage.

Resonant frequency calculation

The resonant frequency is also determined by Eqs. (S7.11) and (S7.12). Note that Eq. (S7.12) has the form of an oscillator equation. The resonant frequency can be calculated from the Taylor series expansion:

$$\frac{C'V_{\mathrm{G}}^2(t)}{2m_{\mathrm{eff}}} - \frac{8ES}{m_{\mathrm{eff}}L}\left(\frac{1}{4}\frac{x^2}{L^2} - 2\frac{q}{L} + \epsilon_0\right)x \approx x_0 - \omega^2 x \tag{S7.13}$$

where x_0 and ω^2 are two expansion factors. Thus we have:

$$\begin{aligned}\omega^2 &= -\frac{\partial}{\partial x}\left(\frac{C'V_{\mathrm{G}}^2(t)}{2m_{\mathrm{eff}}} - \frac{8ES}{m_{\mathrm{eff}}L}\left(\frac{1}{4}\frac{x^2}{L^2} - 2\frac{q}{L} + \epsilon_0\right)x\right) \\ &= \frac{8ES}{m_{\mathrm{eff}}L}\left(\frac{3}{4}\frac{x^2}{L^2} - 2\frac{q}{L} + \epsilon_0\right)\end{aligned} \tag{S7.14}$$

Then Eq. (S7.12) can be approximated as:

$$\frac{\mathrm{d}^2x}{\mathrm{d}t^2} + \omega^2 x \approx x_0 \tag{S7.15}$$

So the resonant frequency can be calculated using:

$$f = \frac{\omega}{2\pi} = \frac{1}{2\pi}\sqrt{\frac{8ES}{m_{\mathrm{eff}}L}\left(\frac{3}{4}\frac{x^2}{L^2} - 2\frac{q}{L} + \epsilon_0\right)} \tag{S7.16}$$

We use Eq. (S7.16) to reproduce the experimental results. The simulated results are shown in

Fig. 4 of the main text. The parameters used in the calculation are based on the experiment. The capacitive characteristics of the device are determined by COMSOL simulations in Section S5, where $C' = 1.1 \times 10^{-9}\ \mathrm{F\,m^{-1}}$. We use $E = 1\ \mathrm{TPa}$ as the Young's modulus of the few-layer graphene [S8]. The length and width of the suspended graphene are measured from scanning electron microscope (SEM) images to be $L = 1.82\ \mu\mathrm{m}$ and $W = 0.52\ \mu\mathrm{m}$, respectively. Using a "regular" model [S3, S9] to fit the tuning curve of the resonant frequency as a function of gate voltage, we estimate that the flake is about 15 layer thick. Since the thickness of a single layer of graphene is 0.34 nm [S10], the cross-sectional area of the graphene flake is $S = 2.74 \times 10^{3}\ \mathrm{nm^{2}}$. To make the simulated results comparable to the experiment, the three free parameters k, c and ϵ_0 are found to be $8.9 \times 10^{2}\ \mathrm{kg\,s^{-2}}$, $5.3 \times 10^{5}\ \mathrm{kg\,s^{-1}}$ and 2.8×10^{-4}, respectively.

Figure S8 shows several simulated parameters as a function of time t during one cycle of a frequency loop. The figure is obtained using a stepping rate of 0.0051 V $\mathrm{s^{-1}}$. From this we can provide an estimate for the elongation $q{\sim}0.8$ nm and the damping force $c\frac{\mathrm{d}q}{\mathrm{d}t}{\sim}10^{-7}$ N.

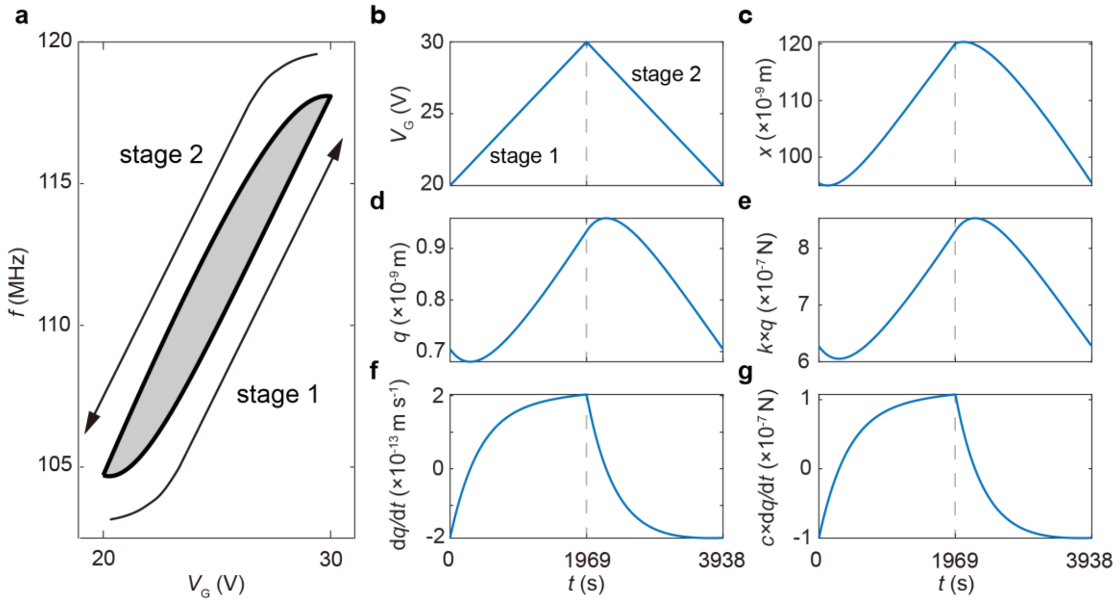

Fig. S8. (a) Schematic of a frequency loop. Simulated time dependence of (b) V_{G}, (c) x, (d) q, (e) $k \times q$, (f) $\mathrm{d}q/\mathrm{d}t$ and (g) $c \times \mathrm{d}q/\mathrm{d}t$ during one cycle of a frequency loop.

S8. Linear relationship between sliding losses and loop area

As with hysteresis loops in other physical systems, the area of our frequency loop is found to be proportional to energy losses incurred during the sliding motion of the graphene flake at the

boundary. It is difficult to directly measure these sliding losses, but they can be estimated numerically within our model. Since $T = kq + c\frac{\mathrm{d}q}{dt}$, sliding losses can be calculated from a counterclockwise loop integral along the frequency loop:

$$Sliding\ Losses = \oint c\frac{\mathrm{d}q}{\mathrm{d}t}\mathrm{d}q \tag{S8.1}$$

The loop area can also be obtained from a similar loop integral:

$$Loop\ Area = -\oint f\mathrm{d}V_\mathrm{G} \tag{S8.2}$$

Figure S9 illustrates how sliding losses and loop area are calculated.

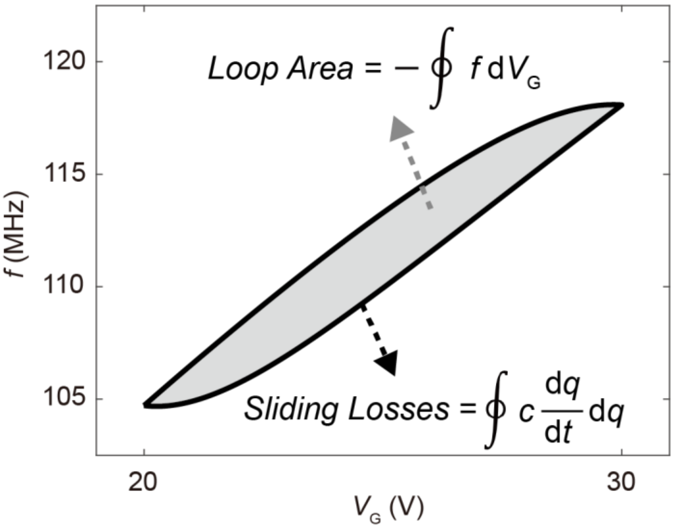

Fig. S9. Calculating sliding losses and loop area.

Based on the parameters for obtaining Fig. 4 in the main text, we calculate different frequency loops by varying the stepping rate and V_G range. We vary the stepping rate from 0.0020 V s^{-1} to 0.0120 V s^{-1}, which encompasses the stepping rate used in the experiment. We find that, for a given V_G range, sliding losses scale linearly with the loop area as the stepping rate is changed (Fig. S10a). When the range of V_G is changed, the linear dependence still holds except for a slight change in the slope (Fig. S10b).

Figure S10a shows sliding losses versus loop area in the case where V_G is cycled within the 20 V to 30 V range. The markers correspond to the stepping rate defined in Fig. 4e of the main text. The slope is found to be 1.01×10^{-24} $\mathrm{kg\ m^2\ s^{-1}\ V^{-1}}$. Thus, for a given frequency loop, we can estimate sliding losses from the loop area using this linear dependence. Taking the case of stepping rate=0.0051 V s^{-1} and V_G range=20 V−30 V (the fourth panel from the left in Fig. 4d of

the main text, marked by the circle) as an example, the sliding losses can be estimated to be 4.83×10^{-17} J.

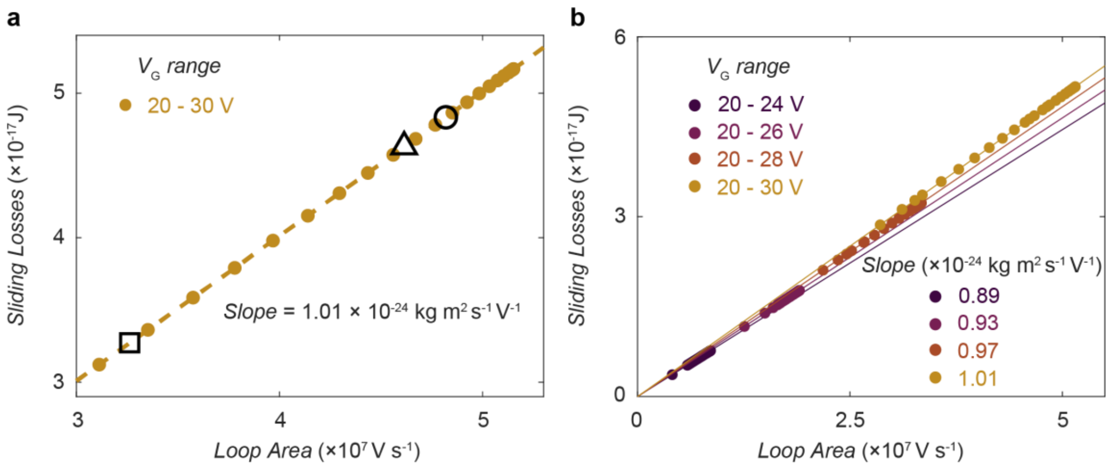

Fig. S10. Linear relationship between sliding losses and loop area, where the data points are extracted from the calculation using different stepping rates for different V_G ranges. (a) Linear dependence for V_G ranging from 20 V to 30 V. (b) When the V_G range is changed, the linear dependence still holds except for a slight change in the slope.

S9. Analytical calculation in the limit of a narrow frequency loop

Our model is described by Eqs. (S7.11), (S7.12), and (S7.16), which we reproduce below for convenience:

$$\frac{\mathrm{d}q}{\mathrm{d}t} = -\left(\frac{k}{c} + 2\frac{ES}{cL}\right)q + \frac{1}{4}\frac{ES}{cL^2}x^2 + \frac{ES}{c}\epsilon_0 \tag{S9.1}$$

$$\frac{\mathrm{d}^2x}{\mathrm{d}t^2} = \frac{c'V_G^2(t)}{2m_{\mathrm{eff}}} - \frac{8ES}{m_{\mathrm{eff}}L}\left(\frac{1}{4}\frac{x^2}{L^2} - 2\frac{q}{L} + \epsilon_0\right)x \tag{S9.2}$$

$$f = \frac{\omega}{2\pi} = \frac{1}{2\pi}\sqrt{\frac{8ES}{m_{\mathrm{eff}}L}\left(\frac{3}{4}\frac{x^2}{L^2} - 2\frac{q}{L} + \epsilon_0\right)} \tag{S9.3}$$

It is difficult to give a general analytical solution to the model. Alternatively, we can provide an analytical solution in the limit of a narrow frequency loop to understand the linear dependence between sliding losses and loop area.

In the experiment, the gate voltage V_G changes linearly in time for both “upward” and “downward” stepping directions:

$$V_G = V_\pm \pm k_1 t \tag{S9.4}$$

where the stepping rate $k_1 > 0$. Plus and minus signs represent “upward” and “downward” directions, respectively. V_+ and V_- are the initial gate voltages for “upward” and “downward”

directions, respectively. We have $V_+ + k_1 t \leq V_-$ and $V_- - k_1 t \geq V_+$.

When the stepping rate k_1 tends to 0, the frequency loop narrows down. Over a short period after the step direction has changed, the variables x and q can be assumed to have a linear dependence on V_G (see the numerical results shown in Figs. S11c and S11d):

$$x = x_\pm + k_2 V_\mathrm{G} = x_\pm + k_2 V_\pm \pm k_1 k_2 t \tag{S9.5}$$

$$q = q_\pm + k_3 V_\mathrm{G} = q_\pm + k_3 V_\pm \pm k_1 k_3 t \tag{S9.6}$$

Since the loop is narrow, we have $\frac{x_- - x_+}{x_- + x_+} \ll 1, \frac{q_- - q_+}{q_- + q_+} \ll 1$.

Figure S11a summarizes the definitions of $V_\pm$, $x_\pm$, $q_\pm$, $f_\pm$, k_2, k_3 and k_4. The loop area of $x(V_\mathrm{G})$, $q(V_\mathrm{G})$ and $f(V_\mathrm{G})$ (see the numerical results shown in Figs. S11b-d) can be approximated by the gray area shown in Fig. S11a, defined by the two vertical lines and the two parallel lines with slopes k_2, k_3, and k_4, respectively. The two vertical lines correspond to $V_\mathrm{G} = V_-$ and $V_\mathrm{G} = V_+$.

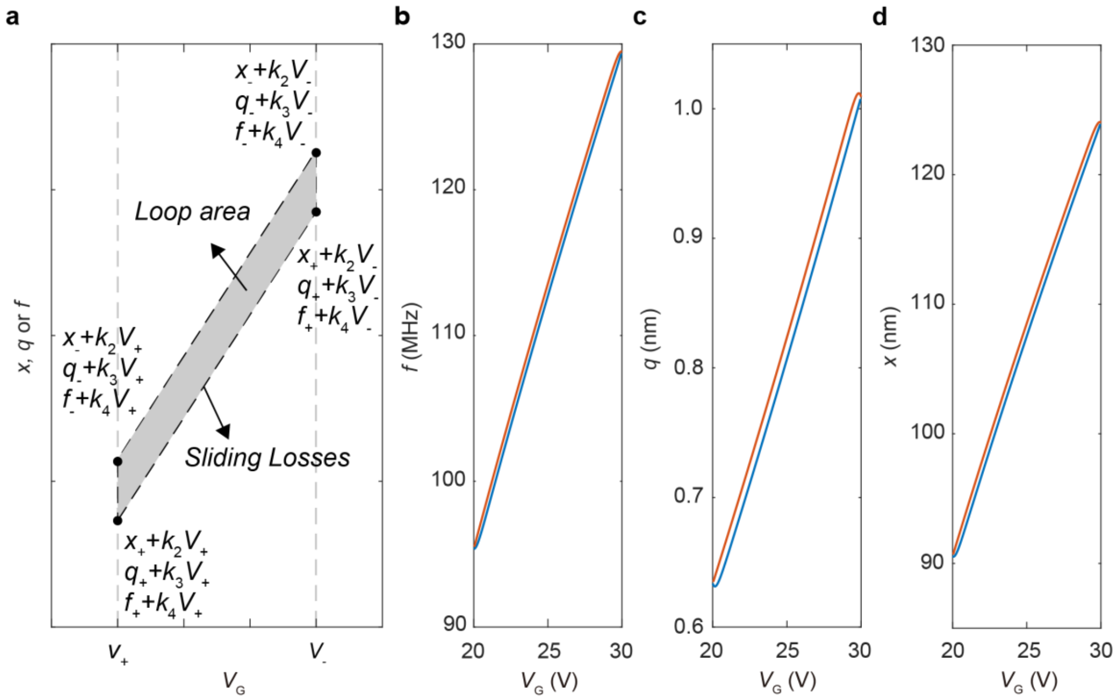

Fig. S11. (a) Schematic depicting the narrow frequency loop approximation. Numerical results for loops of (b) f, (c) q, and (d) x.

Using such a linear approximation in the narrow loop limit, sliding losses during a loop can be estimated as:

$$\mathit{Sliding\ Losses} = \oint c \frac{\mathrm{d}q}{\mathrm{d}t} \mathrm{d}q \approx c k_1 k_3 \oint |\mathrm{d}q| = 2c(V_- - V_+) k_1 k_3^2 \tag{S9.7}$$

Similarly, the relationship between resonant frequency f and V_{G} can also be approximated by a linear dependence (Figs. S11a and S11b):

$$f = f_{\pm} + k_4 V_{\mathrm{G}} \tag{S9.8}$$

where $\frac{f_- - f_+}{f_- + f_+} \ll 1$.

The area of the frequency loop can be estimated as:

$$Loop\ Area = -\oint f \mathrm{d}V_{\mathrm{G}} \approx (f_- - f_+)(V_- - V_+) \tag{S9.9}$$

As a result, we have:

$$\frac{Sliding\ Losses}{Loop\ Area} = \frac{2ck_1k_3^2}{f_- - f_+} \tag{S9.10}$$

Since $\frac{f_- - f_+}{f_- + f_+} \ll 1$, $f_- - f_+$ can be approximated by the differential of Eq. (S9.3). So we have:

$$\mathrm{d}(f) = \frac{1}{2\pi}\sqrt{\frac{8ES}{m_{\mathrm{eff}}L}}\frac{\frac{3}{4}\frac{1}{L^2}\mathrm{d}(x^2) - 2\frac{1}{L}\mathrm{d}q}{2\sqrt{\left(\frac{3x^2}{4L^2} - 2\frac{q}{L} + \epsilon_0\right)}} = \frac{1}{4\pi^2}\frac{8ES}{m_{\mathrm{eff}}L}\frac{1}{2f}\left(\frac{3}{4}\frac{1}{L^2}\mathrm{d}(x^2) - 2\frac{1}{L}\mathrm{d}q\right) \tag{S9.11}$$

Thus, $f_- - f_+$ can be estimated as:

$$f_- - f_+ \approx \frac{ES}{\pi^2 m_{\mathrm{eff}}L}\frac{\frac{3}{4}\frac{1}{L^2}(x_-^2 - x_+^2) - 2\frac{1}{L}(q_- - q_+)}{f} \tag{S9.12}$$

Plugging Eq. (S9.12) into Eq. (S9.10), we obtain:

$$\frac{Sliding\ Losses}{Loop\ Area} = \frac{2\pi^2 ck_1 m_{\mathrm{eff}}L\cdot f}{ES}\frac{k_3^2}{\frac{3}{4}\frac{1}{L^2}(x_-^2 - x_+^2) - 2\frac{1}{L}(q_- - q_+)} \tag{S9.13}$$

Now, we need to calculate k_3, $x_-^2 - x_+^2$ and $q_- - q_+$ from the linearized model. Plugging Eqs. (S9.4), (S9.5) and (S9.6) into Eqs. (S9.1) and (S9.2), we have:

$$+k_1k_3 = -\left(\frac{k}{c} + 2\frac{ES}{cL}\right)(q_+ + k_3V_{\mathrm{G}}) + \frac{1}{4}\frac{ES}{cL^2}(x_+ + k_2V_{\mathrm{G}})^2 + \frac{ES}{c}\epsilon_0 \tag{S9.14}$$

$$-k_1k_3 = -\left(\frac{k}{c} + 2\frac{ES}{cL}\right)(q_- + k_3V_{\mathrm{G}}) + \frac{1}{4}\frac{ES}{cL^2}(x_- + k_2V_{\mathrm{G}})^2 + \frac{ES}{c}\epsilon_0 \tag{S9.15}$$

$$\frac{8ES}{m_{\mathrm{eff}}L}\left(\frac{1}{4}\frac{(x_+ + k_2V_{\mathrm{G}})^2}{L^2} - 2\frac{(q_+ + k_3V_{\mathrm{G}})}{L} + \epsilon_0\right) = \frac{C'V_{\mathrm{G}}^2}{2m_{\mathrm{eff}}}\frac{1}{x_+ + k_2V_{\mathrm{G}}} \tag{S9.16}$$

$$\frac{8ES}{m_{\mathrm{eff}}L}\left(\frac{1}{4}\frac{(x_- + k_2V_{\mathrm{G}})^2}{L^2} - 2\frac{(q_- + k_3V_{\mathrm{G}})}{L} + \epsilon_0\right) = \frac{C'V_{\mathrm{G}}^2}{2m_{\mathrm{eff}}}\frac{1}{x_- + k_2V_{\mathrm{G}}} \tag{S9.17}$$

Subtracting Eq. (S9.14) from Eq. (S9.15) and subtracting Eq. (S9.16) from Eq. (S9.17), and letting $x = \frac{x_- + x_+}{2} + k_2V_{\mathrm{G}}$, $q = \frac{q_- + q_+}{2} + k_3V_{\mathrm{G}}$, $\frac{ES}{kL} = p_1$ and $\frac{C'V_{\mathrm{G}}^2}{8ES} = p_2$, we obtain:

$$(1 + 2p_1)(q_- - q_+) - \frac{1}{2}p_1\frac{x}{L}(x_- - x_+) = \frac{2ck_1k_3}{k} \tag{S9.18}$$

$$\frac{x(x_+ - x_-)}{L^3} - 4\frac{(q_+ - q_-)}{L^2} = p_2\left(\frac{1}{x_+ + k_2V_{\mathrm{G}}} - \frac{1}{x_- + k_2V_{\mathrm{G}}}\right) \tag{S9.19}$$

Because $\frac{1}{x_+ + k_2 V_G} - \frac{1}{x_- + k_2 V_G} \approx \left(\frac{x_- - x_+}{x^2}\right)$, Eq. (S9.19) can be transformed into:

$$\frac{q_- - q_+}{x_- - x_+} = \frac{1}{4}\left(1 + p_2 \left(\frac{x}{L}\right)^{-3}\right)\frac{x}{L} \quad \text{(S9.20)}$$

The parameters E, S, k, L and C' are known. Assuming $V_G \sim 20$ V, we get $p_1 = 1.69$, $p_2 = 2.01 \times 10^{-5}$.

If we assume that each term on the right-hand side of Eq. (S9.2) has the same order of magnitude (which is confirmed by the numerical analysis), then we can estimate the orders of $\frac{x}{L}$ and $\frac{q}{L}$:

$$\frac{x}{L} \sim \left(\frac{C' V_G^2}{8ES}\right)^{\frac{1}{3}} = p_2^{\frac{1}{3}} = 2.72 \times 10^{-2} \quad \text{(S9.21)}$$

$$\frac{q}{L} \sim \left(\frac{C' V_G^2}{8ES}\right)^{\frac{2}{3}} = p_2^{\frac{2}{3}} = 7.39 \times 10^{-4} \quad \text{(S9.22)}$$

k_3 can be estimated as:

$$k_3 = \frac{dq}{dV_G} \sim \frac{q}{V_G} = \frac{q}{L}\frac{L}{V_G} = p_2^{\frac{2}{3}}\frac{L}{V_G} \sim 6.73 \times 10^{-11}\ \text{m V}^{-1} \quad \text{(S9.23)}$$

Plugging Eq. (S9.21) into Eqs. (S9.18) and (S9.2), we have:

$$\frac{q_- - q_+}{x_- - x_+} = \frac{1}{2} p_2^{\frac{1}{3}} \quad \text{(S9.24)}$$

$$(1 + 2p_1)(q_- - q_+) - \frac{1}{2} p_1 p_2^{\frac{1}{3}} (x_- - x_+) = \frac{2ck_1k_3}{k} \quad \text{(S9.25)}$$

So we can solve $x_- - x_+$ and $q_- - q_+$ to be:

$$x_- - x_+ = \frac{4ck_1k_3}{k(1+p_1)p_2^{\frac{1}{3}}} \quad \text{(S9.26)}$$

$$q_- - q_+ = \frac{2ck_1k_3}{k(1+p_1)} \quad \text{(S9.27)}$$

Plugging Eqs. (S9.21), (S9.26) and (S9.27) into Eq. (S9.13), the denominator can be expressed as:

$$\frac{3}{4}\frac{1}{L^2}(x_-^2 - x_+^2) - 2\frac{1}{L}(q_- - q_+) = \frac{2}{L}\left[\frac{3}{4}\frac{x}{L}(x_- - x_+) - (q_- - q_+)\right]$$

$$= \frac{2}{L}\left[\frac{3}{4}p_2^{\frac{1}{3}} - \frac{1}{2}p_2^{\frac{1}{3}}\right](x_- - x_+) = \frac{2}{1+p_1}\frac{ck_1k_3}{kL} \sim 2.1 \times 10^{-5} \quad \text{(S9.28)}$$

Here we let k_1= 0.0013 V s^{-1}, which is the stepping rate in the leftmost panel of Fig. 3a in the main text. Thus, the frequency difference is:

$$f_- - f_+ = \frac{ES}{\pi^2 m_{\text{eff}} L}\frac{\frac{3}{4}\frac{1}{L^2}(x_-^2 - x_+^2) - 2\frac{1}{L}(q_- - q_+)}{f} \sim 5.7 \times 10^6\ \text{Hz} \quad \text{(S9.29)}$$

which is comparable to the frequency difference in the experiment.

Plugging Eq. (S9.28) into Eq. (S9.13), we have:

$$\frac{Sliding\ Losses}{Loop\ Area}=\frac{2\pi^2 ck_1 m_{\rm eff}L\cdot f}{ES}\frac{k_3^2}{\frac{2}{1+p_1}\frac{ck_1k_3}{kL}}$$

$$=\pi^2\frac{1+p_1}{p_1}p_2^{\frac{2}{3}}m_{\rm eff}L^2\cdot f\cdot V_{\rm G}^{-1} \qquad \text{(S9.30)}$$

with $p_1=\frac{ES}{kL}$ and $p_2=\frac{C'V_{\rm G}^2}{8ES}$.

Plugging p_1, p_2, $V_{\rm G}$ and $m_{\rm eff}=5.7\times10^{-18}$ kg, $L=1.82\times10^{-6}$ m, $f{\sim}10^8$ Hz into Eq. (S9.30), we estimate:

$$\frac{Sliding\ Losses}{Loop\ Area}\approx 0.0116\times m_{\rm eff}L^2\cdot f\cdot V_{\rm G}^{-1}{\sim}1.1\times10^{-24}\ {\rm kg\ m^2\ s^{-1}\ V^{-1}} \qquad \text{(S9.31)}$$

which is comparable to the value obtained from numerical simulations ($(0.89{\sim}1.01)\times 10^{-24}\ {\rm kg\ m^2\ s^{-1}\ V^{-1}}$).

The analytical calculations confirm that the loop area is proportional to the sliding induced energy dissipation, with the estimated ratio $\frac{Sliding\ Losses}{Loop\ Area}$ comparable to that obtained from numerical simulations. From the analytical analysis, we find that the ratio $\frac{Sliding\ Losses}{Loop\ Area}$, which is the slope of the linear fit shown in Fig. S10, does not depend on the stepping rate k_1.

S10. Frequency loops measured in an additional device

Figure S12 shows frequency loops measured in an additional device (Device B). The shapes of these loops are similar to those presented in the main text (measured in Device A). Device B and Device A have a similar structure. The suspending length and width of Device B are $L=1.98\ \mu$m and $W=3.24\ \mu$m, respectively. The graphene flake in Device B is about 300 layer thick, thus the cross-sectional area is $S=2.67\times10^5\ {\rm nm}^2$. We also investigate frequency loops under different gate voltage stepping rates. The stepping rates $dV_{\rm G}/dt$ are 0.00316 V s^{-1} (Figs. S12a-b), 0.00467 V s^{-1} (Figs. S12d-e) and 0.0146 V s^{-1} (Figs. S12g-h), respectively. We use the sliding model described in Section S7 to reproduce the experimental results obtained with each stepping rate, as shown in Figs. S12c, S12f, and S12i, respectively. The three free parameters used for Device B are $\epsilon_0=1.4\times10^{-5}$, $k=6.7\times10^4$ kg s^{-2} and $c=4.0\times10^7$ kg s^{-1}. Figure S12k shows the simulated width of the frequency loops $\Delta V_{\rm G}$ as a function of $dV_{\rm G}/dt$, which is in good agreement with experimental results shown in Fig. S12j.

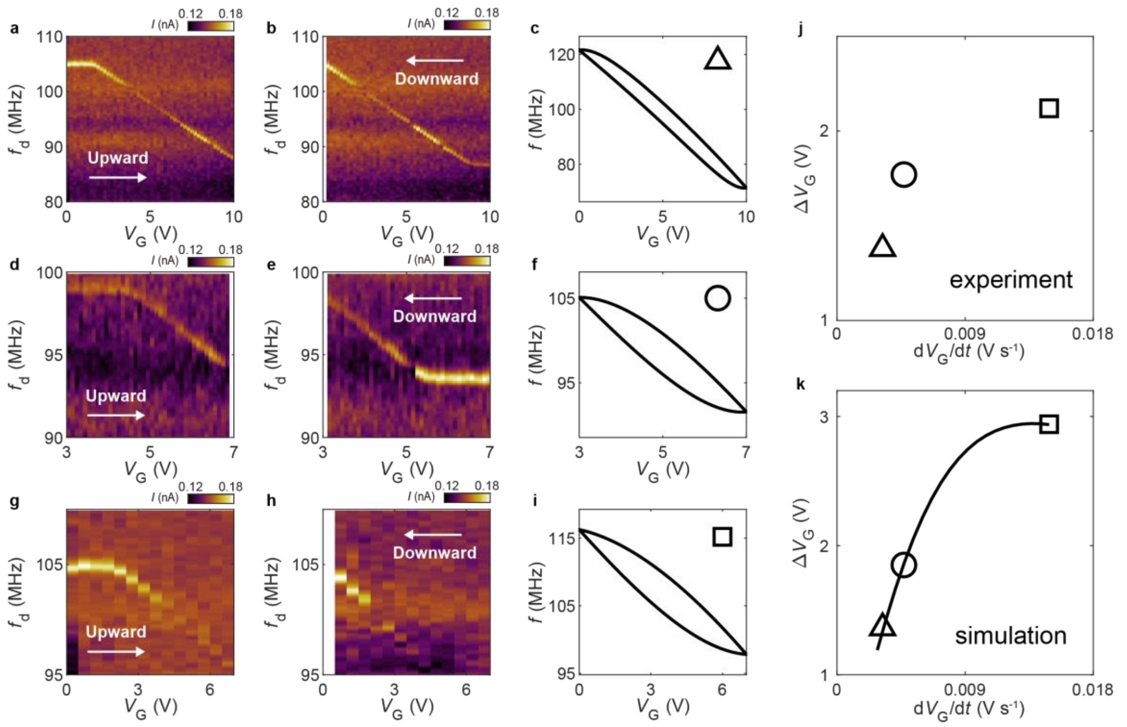

Fig. S12. Resonant frequency loops and their dependence on stepping rate $\mathrm{d}V_{\mathrm{G}}/\mathrm{d}t$ for Device B. The response of the resonator upon increasing/decreasing gate voltage V_{G} is labelled as "upward"/"downward". Frequency loops are measured using the stepping rates (a-b) 0.00316 V s^{-1}, (d-e) 0.00467 V s^{-1}, and (g-h) 0.0146 V s^{-1}, respectively, with simulated results correspondingly shown in (c), (f), and (i). (j) ((k)) shows the measured (simulated) width of frequency loops obtained under different stepping rates.

Table S1 summarizes the parameters of Device A (presented in the main text) and those of Device B (presented in Section S10). Note that the spring constant k and damping coefficient c of Device B are two orders of magnitude larger than those of Device A owing to the larger thickness of Device B. However, the estimated sliding losses per unit cell of both devices are close to $\sim 10^{-20}$ J obtained from friction force microscopy experiments [S11]. This suggests that the area within the loop drawn by the resonant frequency in the space spanned by frequency and gate voltage is a measure of the friction energy dissipated as the membrane slides back and forth on its support (as discussed in Sections S8 and S9). In addition, estimates for the frictional shear stress τ in both devices are similar. The dashpot in our sliding model captures the effect of friction at the nanoscale well. Moreover, although the spring constant k of Device A and that of Device B differ by two orders of magnitude, we find that estimates of the normalized spring constant, kL/ES, are

close for both devices. The physical meaning of the normalized spring constant is revealed in Section S12. The interesting dependence of c and k on geometric parameters of the resonator calls for systematic studies with devices of various dimensions.

**Physical Quantity**	**Symbol**	**Device A**	**Device B**
Length	L	1.82×10^{-6} m	1.98×10^{-6} m
Width	W	0.52×10^{-6} m	3.24×10^{-6} m
Cross-sectional area	S	2.74×10^{-15} m^2	2.67×10^{-13} m^2
Effective mass	m_{eff}	5.7×10^{-18} kg	6.08×10^{-16} kg
Derivative of capacitance	C'	1.1×10^{-9} F m^{-1}	1.25×10^{-9} F m^{-1}
Built-in strain	ϵ_0	2.8×10^{-4}	1.4×10^{-5}
Spring constant	k	8.9×10^{2} kg s^{-2}	6.7×10^{4} kg s^{-2}
Damping coefficient	c	5.3×10^{5} kg s^{-1}	4.0×10^{7} kg s^{-1}
Sliding losses	$\oint c \frac{\mathrm{d}q}{\mathrm{d}t} \mathrm{d}q$	4.83×10^{-17} J	6.22×10^{-17} J
Sliding losses per unit cell	$\frac{\oint c \frac{\mathrm{d}q}{\mathrm{d}t} \mathrm{d}q}{\text{number of cells}}$	6.09×10^{-21} J	6.72×10^{-21} J
Frictional shear stress	τ	0.20 MPa	0.17 MPa
Normalized spring constant	kL/ES	0.59	0.50

Table S1. List of parameters for Device A and Device B.

S11. Absence of frequency loop in "regular" devices

Figure S13a shows f as a function of V_{G} measured in a "regular" device that does not show frequency loops. Although the "regular" device also consists of three coupled resonators (similar to Device A and Device B, see Section 12 for details), its frequency tuning spectrum is symmetric (Fig. S13a) and frequency loops are not observed (Figs. S13b and S13c).

We have observed frequency loops in 2 out of the 8 devices we fabricated. The reason why frequency loops are observed only in some devices, even though all the devices look structurally similar, is currently unknown. However, we have noticed that the two-terminal resistances of devices exhibiting frequency loops (Device A and Device B) are two orders of magnitude lower than those of devices that do not show any loop. This fact, admittedly based on a limited number of devices, points to a difference among devices regarding the cleanliness of interfaces between graphene and metal electrodes. Namely, we believe that, in the case of Device A and Device B,

such interfaces may be less contaminated with adsorbates than in the case of the other devices. In turn, a lesser amount of adsorbates may favor the sliding of graphene on electrodes.

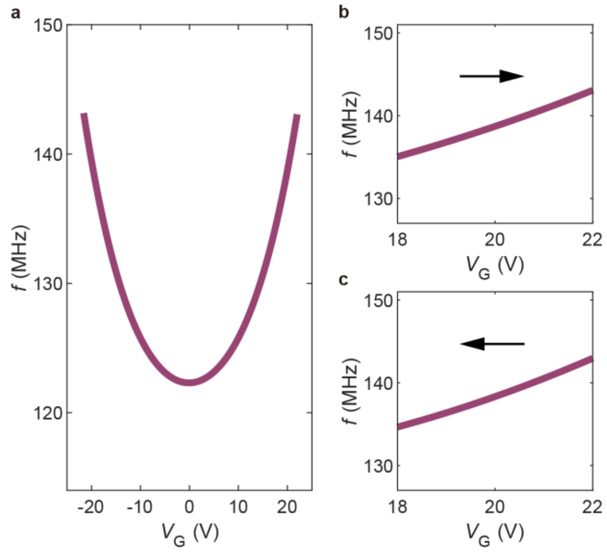

Fig. S13. (a) Resonant frequency f as a function of V_{G} showing a symmetric gate voltage dependence. (b) ((c)) f as a function of increasing (decreasing) V_{G}. No frequency loop is observed. The data are measured in a "regular" device.

This picture is supported by our estimates of the frictional shear stress τ in Device A, $\tau = 0.20$ MPa, and in Device B, $\tau = 0.17$ MPa (see Table S1). These estimates are lower than those obtained in experiments where metal nanoparticles are pushed along the adsorbate-coated surface of a graphite crystal using the tip of an atomic force microscope probe [S12, S13]. There, τ ranges from several to hundreds of MPa. Such large values for τ are attributed to the presence of interfacial adsorbates [S13, S14]. Therefore, our smaller estimates for τ may indicate clean interfaces, favoring graphene sliding.

We emphasize, however, that the cleanliness of interfaces is currently difficult for us to control. Even though the dry transfer process [S15] we employ to deposit graphene onto electrodes is generally viewed as a clean fabrication technique, it is not free of fabrication residues, nor does it keep hydrocarbon molecules in the air from adsorbing on the electrodes or on the graphene flake itself. Even in the absence of contaminants, structural details of the interfaces are likely to vary from one device to the next since our metal electrodes are amorphous. A better control of the quality of interfaces may improve the yield of devices showing frequency loops.

Clearly, our work invites further research on the control of interfaces between graphene and electrodes. For example, 2-D mechanical resonators held by atomically flat supports (instead of amorphous metal electrodes) would be interesting systems to study. In those systems, the pick-up transfer technique [S16] can be used to minimize contamination and keep interfaces pristine. In this way, ultralow friction, or superlubricity [S17, S18], between atomically smooth surfaces may greatly facilitate the sliding of the membrane, thereby conferring resonators unusual mechanical properties.

S12. Physical origin of spring constant *k*

Extended version of the sliding model

In the sliding model, the most important parameters are the damping coefficient c of the dashpot and the spring constant k of the spring. As discussed in Sections S8, S9, and S10, the physical origin of the dashpot, that is, of the damping coefficient c, is the sliding-induced friction between graphene and its supporting substrate. Meanwhile, the spring in our model makes sliding reversible. In this section, we propose a physical origin for the spring and for its constant k by considering the influence of the neighboring resonators on both sides of the measured resonator. This is an extended version of the sliding model.

Below, we consider Device A (presented in the main text). Figure S14a is a colorized SEM image of Device A (see also Fig. 5a). A uniform graphene ribbon is suspended over pre-patterned trenches, forming three graphene resonators coupled in series and labelled as R_{L}, R_{M}, and R_{R}. The resonator investigated in the main text is the middle one, labelled as R_{M}. As shown in Fig. S14b, we use the sliding model (as discussed in Section S7) to account for the experimental results, in which a dashpot and a spring (as shown in Fig. S14c) are attached to each of the two supporting edges of the membrane to model the sliding motion.

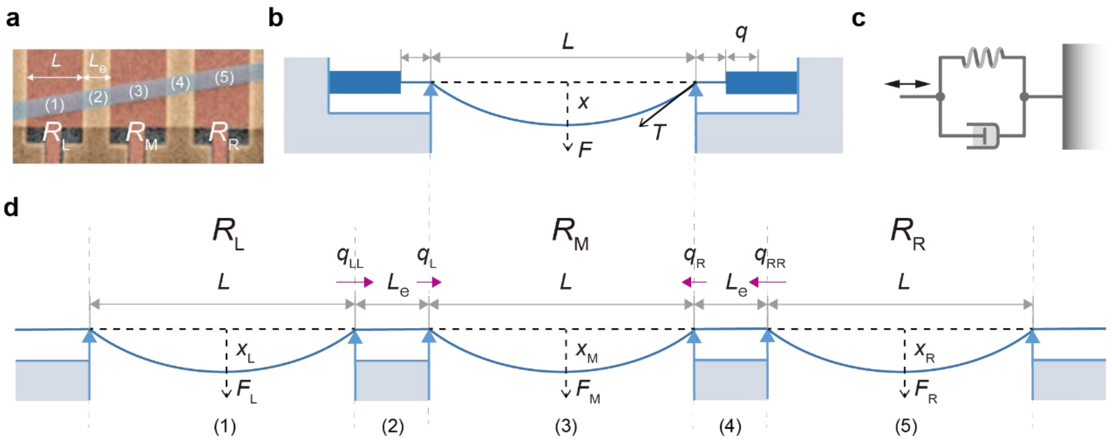

Fig. S14. (a) Colorized SEM image of Device A, showing three resonators coupled in series and labelled as R_{L}, R_{M}, and R_{R}. The middle resonator, R_{M}, is the resonator investigated in the main text. (b) Schematic illustrating the sliding model from Section S7. (c) Schematic of the viscoelastic connector, consisting of a spring and a dashpot, that we use to describe sliding. (d) Schematic depicting the extended version of our sliding model, which includes the effect of the neighboring resonators.

Now we take the neighboring resonators (R_{L} and R_{R}) into consideration, as illustrated in Fig. S14d. The parts labelled as (1), (3), and (5) are the regions where the graphene ribbon is suspended to form resonators R_{L}, R_{M}, and R_{R}, respectively. The parts (2) and (4) are metal contacts where sliding occurs. When extra length of membrane is fed into the suspended area (3) due to sliding, it not only modifies the resonant frequency of R_{M}, as already discussed in the sliding model, it also strains the neighboring resonators R_{L} and R_{R}. As a consequence, the neighboring resonators provide restoring forces. We propose that this effect of the neighboring resonators may be the physical origin of the spring and of its constant k (as illustrated in Figs. 5b and 5c).

Figures S14a and S14d show the structure of the device. L is the length of the trench and L_{e} is the length of the metal contact part. With reference to Fig. S14d, the sliding distance of the membrane on the left of R_{M} (on the right of R_{L}) is q_{L} (q_{LL}). q_{R} and q_{RR} are defined in a similar way. Note that q_{L} and q_{R} are sliding distances at the two boundaries of R_{M}, respectively, and as such they directly correspond to the deformation of the viscoelastic connector q in the sliding model from Section S7. As in Eq. (S7.6), the tension T_{i} (i=1-5) for each part can be written

as:

$$T_1 = ES\left(\frac{1}{4}\frac{x_{\mathrm{L}}^2}{L^2} + \frac{q_{\mathrm{LL}}}{L} + \epsilon_0\right) \quad \text{(S12.1)}$$

$$T_2 = ES\left(\frac{q_{\mathrm{L}} - q_{\mathrm{LL}}}{L_{\mathrm{e}}} + \epsilon_0\right) \quad \text{(S12.2)}$$

$$T_3 = ES\left(\frac{1}{4}\frac{x_{\mathrm{M}}^2}{L^2} - \frac{q_{\mathrm{L}}}{L} - \frac{q_{\mathrm{R}}}{L} + \epsilon_0\right) \quad \text{(S12.3)}$$

$$T_4 = ES\left(\frac{q_{\mathrm{R}} - q_{\mathrm{RR}}}{L_{\mathrm{e}}} + \epsilon_0\right) \quad \text{(S12.4)}$$

$$T_5 = ES\left(\frac{1}{4}\frac{x_{\mathrm{R}}^2}{L^2} + \frac{q_{\mathrm{RR}}}{L} + \epsilon_0\right) \quad \text{(S12.5)}$$

where x_{L}, x_{M} and x_{R} are the maximum static displacements of R_{L}, R_{M}, and R_{R}, respectively. E, S, and ϵ_0 are the Young's modulus, the cross-sectional area, and the built-in strain, which are assumed to be the same in all five parts since they come from the same graphene ribbon.

Here, for simplicity we consider the case of static equilibrium where the influences of vibrations and viscosity are absent. We show that this assumption is sufficient to reveal the physical origin of the spring constant k in the original sliding model. Accordingly, all five parts are subjected to the same tension:

$$T_{1,2,3,4,5} = T \quad \text{(S12.6)}$$

Since R_{L}, R_{M}, and R_{R} can be individually addressed using their gates, we apply Eq. (S7.9) to R_{M}, R_{L}, and R_{R}, and obtain the following conditions for static equilibrium:

$$8T\frac{x_{\mathrm{M}}}{L} = F_{\mathrm{M}}(V_{\mathrm{M}}) \quad \text{(S12.7)}$$

$$8T\frac{x_{\mathrm{L}}}{L} = F_{\mathrm{L}}(V_{\mathrm{L}}) \quad \text{(S12.8)}$$

$$8T\frac{x_{\mathrm{R}}}{L} = F_{\mathrm{R}}(V_{\mathrm{R}}) \quad \text{(S12.9)}$$

where $F_{\mathrm{i}} = \frac{1}{2}C_{\mathrm{i}}'V_{\mathrm{i}}^2$, (i=L, M, R). C_{i}' and V_{i} are the first derivatives of the capacitance of and the gate voltages applied to R_{L}, R_{M}, and R_{R}, respectively.

Using Eqs. (S12.1)-(S12.9), we have:

$$\left(\frac{T}{ES\epsilon_0}\right)^3 - \left(\frac{T}{ES\epsilon_0}\right)^2 - \frac{1}{256\epsilon_0^3E^2S^2}\frac{L}{L+2L_{\mathrm{eff}}}\left(\frac{1}{4}{C_{\mathrm{M}}'}^2V_{\mathrm{M}}^4 + \frac{1}{4}{C_{\mathrm{L}}'}^2V_{\mathrm{L}}^4 + \frac{1}{4}{C_{\mathrm{R}}'}^2V_{\mathrm{R}}^4\right) = 0 \quad \text{(S12.10)}$$

where $L_{\mathrm{eff}} = L + L_{\mathrm{e}}$. We can calculate the tension T by solving this equation. Other parameters are readily obtained as well:

$$x_{\mathrm{L,M,R}} = \frac{F_{\mathrm{L,M,R}}L}{8T} \quad \text{(S12.11)}$$

$$q_{\mathrm{L,R}} = -\frac{1}{4}\frac{x_{\mathrm{L,R}}^2}{L} + \frac{TL_{\mathrm{eff}}}{ES} - L_{\mathrm{eff}}\epsilon_0 \tag{S12.12}$$

The resonant frequency f_{M} of the middle resonator can be calculated as:

$$f_{\mathrm{M}} = \frac{1}{2\pi}\sqrt{\frac{8ES}{m_{\mathrm{eff}}L}\left(\frac{3}{4}\frac{x_{\mathrm{M}}^2}{L^2} - \frac{q_{\mathrm{L}}}{L} - \frac{q_{\mathrm{R}}}{L} + \epsilon_0\right)} \tag{S12.13}$$

which simplifies to Eq. (S7.16) if $q_{\mathrm{L}} = q_{\mathrm{R}} = q$.

Finally, we derive the spring constant k using our extended model. As in Eq. (S7.7), the spring constant $k_{\mathrm{L,R}}$ is defined using the relationship between the tension T and the sliding distance $q_{\mathrm{L,R}}$ on the left and on the right of R_{M}:

$$k_{\mathrm{L}} = \left(\frac{\partial T}{\partial q_{\mathrm{L}}}\right)_{V_{\mathrm{L,R}}} \tag{S12.14}$$

$$k_{\mathrm{R}} = \left(\frac{\partial T}{\partial q_{\mathrm{R}}}\right)_{V_{\mathrm{L,R}}} \tag{S12.15}$$

Plugging Eq. (S12.1) into Eq. (S12.2), we have:

$$T = ES\left(\frac{1}{4}\frac{x_L^2}{L_{\mathrm{eff}}L} + \frac{q_L}{L_{\mathrm{eff}}} + \epsilon_0\right) \tag{S12.16}$$

Differentiating both sides of Eq. (S12.16), we have:

$$k_{\mathrm{L}} = \left(\frac{\partial T}{\partial q_{\mathrm{L}}}\right)_{V_{\mathrm{L,R}}} = \frac{L}{L_{\mathrm{eff}}}\frac{ES}{L}\left(1 + \frac{1}{4L}\left(\frac{\partial x_{\mathrm{L}}^2}{\partial q_{\mathrm{L}}}\right)_{V_{\mathrm{L,R}}}\right) \tag{S12.17}$$

Similarly, from Eqs. (S12.4) and (S12.5), we have:

$$k_{\mathrm{R}} = \left(\frac{\partial T}{\partial q_{\mathrm{R}}}\right)_{V_{\mathrm{L,R}}} = \frac{L}{L_{\mathrm{eff}}}\frac{ES}{L}\left(1 + \frac{1}{4L}\left(\frac{\partial x_{\mathrm{R}}^2}{\partial q_{\mathrm{R}}}\right)_{V_{\mathrm{L,R}}}\right) \tag{S12.18}$$

Using Eqs. (S12.8) and (S12.9), we obtain:

$$\frac{1}{4L}\left(\frac{\partial x_{\mathrm{L,R}}^2}{\partial q_{\mathrm{L,R}}}\right)_{V_{\mathrm{L,R}}} = \frac{L}{256}\left(\frac{\partial \frac{F_{\mathrm{L,R}}^2}{T^2}}{\partial q_{\mathrm{L,R}}}\right)_{V_{\mathrm{L,R}}} = \frac{LF_{\mathrm{L,R}}^2}{256}\left(\frac{\partial \frac{1}{T^2}}{\partial q_{\mathrm{L,R}}}\right)_{V_{\mathrm{L,R}}}$$

$$= -\frac{LF_{\mathrm{L,R}}^2}{128T^3}\left(\frac{\partial T}{\partial q_{\mathrm{L,R}}}\right)_{V_{\mathrm{L,R}}} = -\frac{LF_{\mathrm{L,R}}^2}{128T^3}k_{\mathrm{L,R}} \tag{S12.19}$$

Plugging Eq. (S12.19) into Eqs. (S12.17) and (S12.18), the spring constant $k_{\mathrm{L,R}}$ is:

$$k_{\mathrm{L,R}} = \frac{ES}{L}\frac{L}{L_{\mathrm{eff}}}\left(1 + \frac{L}{L_{\mathrm{eff}}}\frac{ESF_{\mathrm{L,R}}^2}{128T^3}\right)^{-1} \tag{S12.20}$$

If the gate voltage $V_{\mathrm{L,R}} = 0$, that is, $F_{\mathrm{L,R}} = 0$, which is the case for Device A, we have:

$$\frac{k_{\mathrm{L,R}}L}{ES} = \frac{L}{L_{\mathrm{eff}}} = \frac{1}{1 + L_{\mathrm{e}}/L} \tag{S12.21}$$

According to the SEM image, $L = 1.82 \times 10^{-6}$ m and $L_{\mathrm{e}} = 0.95 \times 10^{-6}$ m. Therefore, the dimensionless quantity $\frac{k_{\mathrm{L,R}}L}{ES}$, which can be regarded as a normalized spring constant, is estimated

to be 0.66 within the extended model. This result is close to the value for $\frac{kL}{ES}$ (0.59 for Device A and 0.50 for Device B) obtained within the original sliding model (see also Table S1).

Gate dependence

If nonzero V_L and V_R are applied, we can investigate the gate dependence of $k_\mathrm{L,R}$ numerically using Eq. (S12.20). Figure S15 shows how $k_\mathrm{L,R}$ of Device A and Device B change as a function of V_L and V_R, respectively. The gate voltage V_M for obtaining Figs. S15a and S15b (Figs. S15c and S15d) is 20 V (5 V), which is the typical value for Device A (Device B). For both devices, k_L (k_R) decreases when increasing V_L (V_R) and is almost independent of V_R (V_L). In the experiments, V_L and V_R are kept at 0 for Device A (marked by the red stars in Figs. S15a and S15b). At this point, $k_\mathrm{L} = k_\mathrm{R} = 989\ \mathrm{kg\,s^{-2}}$, which is close to the value of $890\ \mathrm{kg\,s^{-2}}$ estimated within the original sliding model. For Device B, the working point (marked by the red stars in Figs. S15c and S15d) is at $V_\mathrm{L} = 0$, $V_\mathrm{R} = 20\ \mathrm{V}$, resulting in $k_\mathrm{L} = 8.99 \times 10^4\ \mathrm{kg\,s^{-2}}$ and $k_\mathrm{R} = 1.90 \times 10^4\ \mathrm{kg\,s^{-2}}$. The averaged value is $5.45 \times 10^4\ \mathrm{kg\,s^{-2}}$, which is also close to the estimated value of $6.7 \times 10^4\ \mathrm{kg\,s^{-2}}$ obtained within the original sliding model.

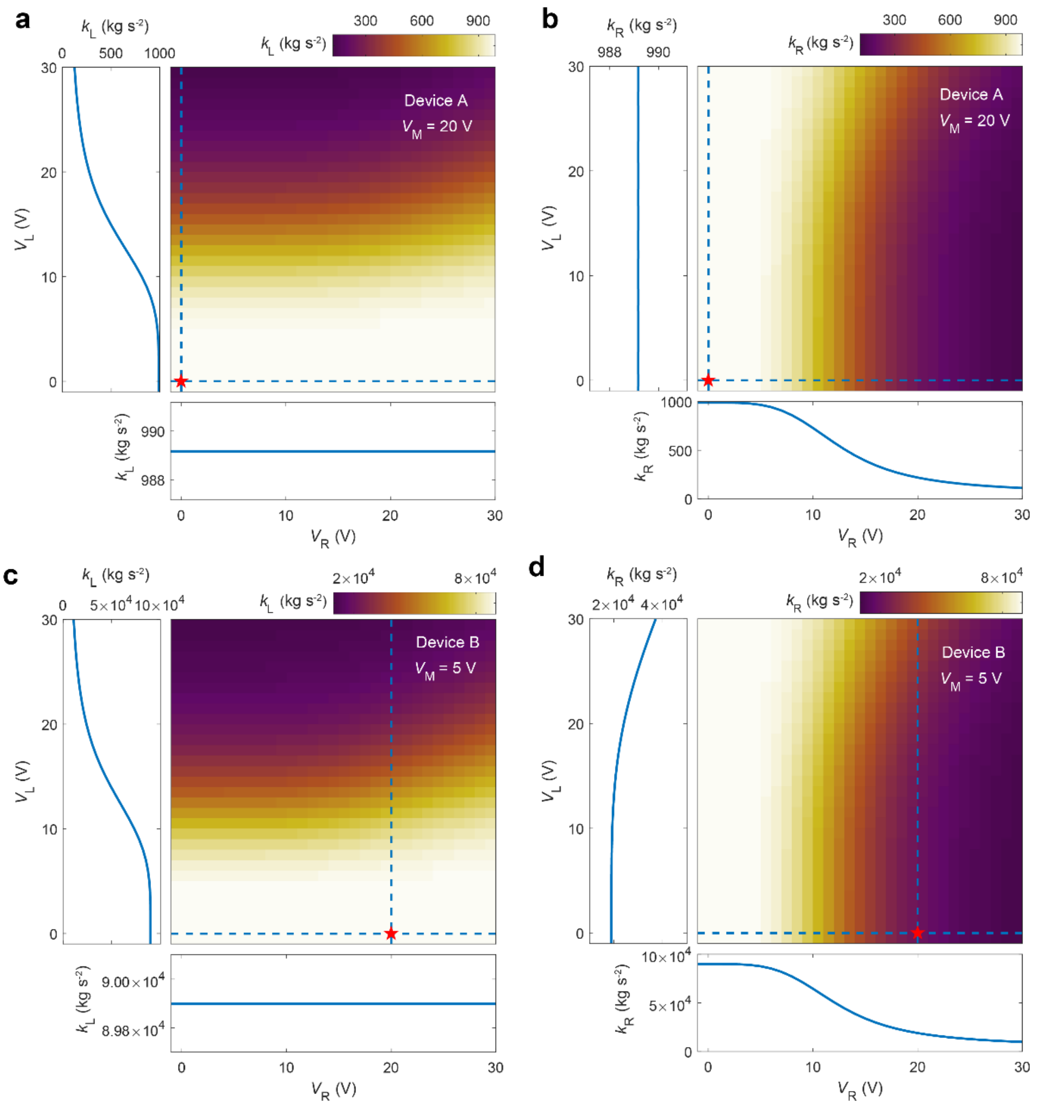

Fig. S15. (a, b) $k_{L,R}$ as a function of V_L and V_R for $V_M = 20$ V and based on the parameters of Device A. (c, d) $k_{L,R}$ as a function of V_L and V_R for $V_M = 5$ V and based on the parameters of Device B. Red stars mark the working points in the experiments.

Such an effect of $V_{L,R}$ on spring constants can also be revealed by tuning the resonant frequency of the middle resonator R_M with gate voltage. Figure S16a shows the measured resonant frequency f_M of R_M as a function of V_M under different V_R. The data are measured in Device A with $V_L = 0$ V and in a separate cooldown from that of the main text. In addition to being tuned by its own gate voltage V_M, the resonant frequency of R_M can also be influenced by gate voltage V_R applied to its neighboring resonator R_R. For a given V_M, decreasing V_R results in a lower resonant frequency f_M. Such a behavior can be qualitatively explained by our extended version of the sliding model. Assuming that all three resonators have the same geometry and

built-in strain, we numerically calculate gate tuning spectra of f_{M} under different V_{R} using Eq. (S12.13). As shown in Fig. S16b, (i) the calculated f_{M} can be tuned by varying V_{R}, and (ii) this tuning is nonlinear in V_{R}. Both effects (i) and (ii) are also observed in the experiment (Fig. S16a).

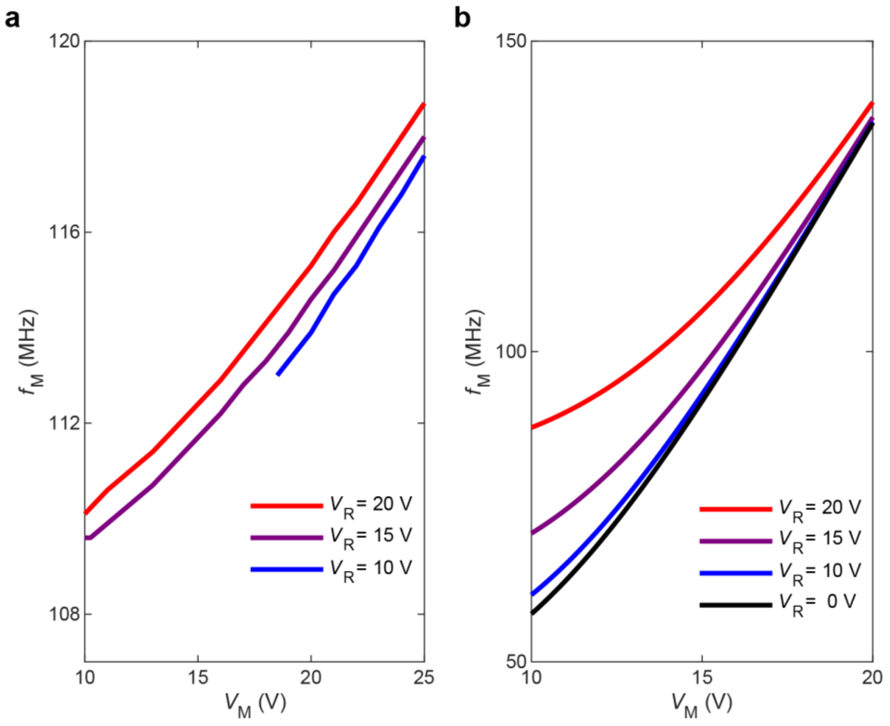

Fig. S16. (a) Measured and (b) calculated resonant frequency of R_{M} as a function of V_{M} under different V_{R} with $V_{\mathrm{L}} = 0$. The experimental data are obtained from Device A and the calculation is done using the extended sliding model based on the parameters of Device A.

In conclusion, according to our extended version of the sliding model, neighboring resonators provide restoring forces as the membrane slides. We propose that this effect is the physical origin of the spring and of its constant k in our sliding model, and that it may explain the reversibility of sliding inferred from the experiments.

References

S1. Migliato Marega, G. et al. Logic-in-memory based on an atomically thin semiconductor. *Nature* **587**, 72-77 (2020).

S2. Gouttenoire, V. et al. Digital and FM Demodulation of a Doubly Clamped Single-Walled Carbon-Nanotube Oscillator: Towards a Nanotube Cell Phone. *Small* **6**, 1060-1065 (2010).

S3. Eichler, A. et al. Nonlinear damping in mechanical resonators made from carbon nanotubes and graphene. *Nat. Nanotechnol.* **6**, 339-342 (2011).

S4. Lifshitz, R. & Cross, M.C. *Nonlinear Dynamics of Nanomechanical and Micromechanical Resonators* in Reviews of Nonlinear Dynamics and Complexity, Vol. 1. (ed. H.G. Schuster) 1-52 (Wiley-VCH, 2008).

S5. Verbiest, G.J. et al. Detecting Ultrasound Vibrations with Graphene Resonators. *Nano Lett.* **18**, 5132-5137 (2018).

S6. Knauss, W.G., Emri, I. & Lu, H. *Mechanics of Polymers: Viscoelasticity* in Springer Handbook of Experimental Solid Mechanics. (eds. J. Sharpe & W. N.) 49-95 (Springer, 2008).

S7. Ferrari, P.F., Kim, S. & van der Zande, A.M. Dissipation from Interlayer Friction in Graphene Nanoelectromechanical Resonators. *Nano Lett.* **21**, 8058-8065 (2021).

S8. Lee, C. et al. Measurement of the Elastic Properties and Intrinsic Strength of Monolayer Graphene. *Science* **321**, 385-388 (2008).

S9. Morell, N. et al. High Quality Factor Mechanical Resonators Based on WSe_2 Monolayers. *Nano Lett.* **16**, 5102-5108 (2016).

S10. Castro Neto, A. H. et al. The electronic properties of graphene. *Rev. Mod. Phys.* **81**, 109 (2009).

S11. Lee, C. et al. Frictional Characteristics of Atomically Thin Sheets. *Science* **328**, 76-80 (2010).

S12. Ritter, C., Heyde, M., Stegemann, B., Rademann, K. & Schwarz, U.D. Contact-area dependence of frictional forces: Moving adsorbed antimony nanoparticles. *Phys. Rev. B* **71**, 085405 (2005).

S13. Dietzel, D. et al. Frictional Duality Observed during Nanoparticle Sliding. *Phys. Rev. Lett.* **101**, 125505 (2008).

S14. Dietzel, D., Feldmann, M., Schwarz, U.D., Fuchs, H. & Schirmeisen, A. Scaling Laws of Structural Lubricity. *Phys. Rev. Lett.* **111**, 235502 (2013).

S15. Castellanos-Gomez, A. et al. Deterministic transfer of two-dimensional materials by all-dry viscoelastic stamping. *2D Mater.* **1**, 011002 (2014).

S16. Wang, L. et al. One-Dimensional Electrical Contact to a Two-Dimensional Material. *Science* **342**, 614-617 (2013).

S17. Kawai, S. et al. Superlubricity of graphene nanoribbons on gold surfaces. *Science* **351**, 957-961 (2016).

S18. Hod, O., Meyer, E., Zheng, Q. & Urbakh, M. Structural superlubricity and ultralow friction across the length scales. *Nature* **563**, 485-492 (2018).
